# Choroidal and Retinal Blood Flow Changes Following Vitrectomy in Two Cases of Postoperative Endophthalmitis

**DOI:** 10.7759/cureus.77006

**Published:** 2025-01-06

**Authors:** Yuki Otsuka, Takatoshi Maeno, Ryuya Hashimoto

**Affiliations:** 1 Ophthalmology, Toho University Sakura Medical Center, Sakura, JPN

**Keywords:** choroid, endophthalmitis, laser speckle flowgraphy, ocular blood flow, pars plana vitrectomy (ppv), retina disease, retina

## Abstract

Postoperative endophthalmitis is a rare but potentially vision-threatening complication following intraocular surgery. We report two cases (one acute onset and one delayed onset) of postoperative endophthalmitis treated with vitrectomy, in which ocular circulation was quantitatively monitored using laser speckle flowgraphy (LSFG). Both cases presented with vitreous opacity, hypopyon, and markedly reduced visual acuity. LSFG measurements demonstrated progressive improvement in both choroidal and retinal blood flow parameters after treatment, accompanied by a reduction in choroidal thickness. These circulatory changes correlated with visual recovery following treatment. Our findings suggest that LSFG may serve as a useful tool for monitoring therapeutic response and predicting visual outcomes in postoperative endophthalmitis.

## Introduction

Postoperative endophthalmitis is a rare but severe complication characterized by infection within intraocular cavities, such as the aqueous or vitreous humor [1]. Among the various types of endophthalmitis, postoperative endophthalmitis is the most prevalent, often occurring as a complication following intraocular surgeries, such as cataract extraction or vitrectomy [2]. In cases of severe postoperative endophthalmitis with significant vision loss, vitrectomy is the preferred treatment option because of its ability to remove infectious material and inflammatory debris [3]. Additionally, the empirical administration of broad-spectrum antibiotics is critical for achieving favorable clinical outcomes [1]. Although the role of adjunctive steroid therapy is debated, it has shown potential benefits in reducing inflammation [4].

Despite the various factors influencing post-treatment clinical outcomes, limited data exist on the changes in retinal and choroidal structures and circulation during the management of postoperative endophthalmitis [5]. Ischemic insults can lead to cumulative retinal cell loss and dysfunction [6]. The choroid plays a critical role in maintaining retinal integrity, and abnormalities in its vasculature can lead to photoreceptor dysfunction and cell death [7,8].

Therefore, assessment of retinal and choroidal structures and circulation is crucial for predicting visual outcomes after endophthalmitis surgery. However, few studies have evaluated these parameters after the treatment of postoperative endophthalmitis [9]. Laser speckle flowgraphy (LSFG-NAVI, Softcare Ltd. Fukuoka, Japan) is a noninvasive imaging technique that allows the quantification of blood flow in the optic nerve head (ONH), retina, and choroid [10-12] using the laser speckle phenomenon. When laser light interacts with a diffusing surface, it produces a speckle pattern caused by the interference of scattered coherent light. The dynamics of this pattern fluctuate in response to the movement of red blood cells, with faster changes indicating higher blood flow [12]. These changes are quantified as the mean blur rate (MBR), where a higher MBR corresponds to increased blood flow velocity. Although LSFG has been extensively applied in other ocular diseases, its use in the context of postoperative endophthalmitis has not been reported.

This report presents two cases of postoperative endophthalmitis in which LSFG was used to monitor choroidal blood flow (CBF) and retinal blood flow dynamics following vitrectomy. To our knowledge, this is the first report to document such findings, providing insights into the recovery of ocular circulation and its implications for visual prognosis.

## Case presentation

Case 1

**Figure 1:**
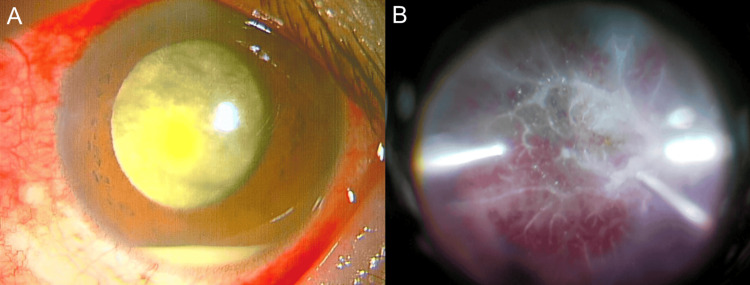
Clinical findings of postoperative endophthalmitis in Case 1 (A) Anterior segment photograph showing dense vitreous opacity with hypopyon and fibrin membrane. (B) Intraoperative fundus photograph showing severe retinal hemorrhages and retinal vascular attenuation.

A 56-year-old Japanese man presented with complaints of worsening visual acuity in his left eye three days after undergoing vitrectomy and cataract surgery for an epiretinal membrane. On initial examination, his best-corrected visual acuity (BCVA) was "hand motions" at 30 cm, and the intraocular pressure (IOP) in his left eye was 13 mmHg. Slit-lamp examination revealed ciliary hyperemia, corneal edema, Descemet's membrane folds, anterior chamber opacities, and an intraocular lens. The fundus view was obscured due to vitreous opacity (Figure 1A). Acute postoperative endophthalmitis was diagnosed based on the clinical course and examination findings.

Emergency vitrectomy revealed vitreous opacities, fibrovascular membranes, and a retinal hemorrhage (Figure 1B). A culture of the vitreous fluid obtained during the operation revealed the presence of methicillin-resistant *Staphylococcus epidermidis*. Postoperatively, the patient received intravenous broad-spectrum antibiotics (imipenem/cilastatin 1 g twice daily) for one week. Additionally, antibiotic eye drops (moxifloxacin/cefepime) were administered for three months, and an antibiotic ointment (ofloxacin) was applied for one month. Oral prednisolone (20 mg/day) was tapered off over two weeks, and anti-inflammatory eye drops (betamethasone/bromfenac) were administered for three months.

One week postoperatively, the patient's BCVA was 20/67. Fundus examination revealed a retinal hemorrhage, vitreous opacity, and fibrovascular membrane. Optical coherence tomography (OCT) imaging was performed using the Spectralis OCT (Heidelberg Engineering, Heidelberg, Germany), a spectral-domain OCT system equipped with enhanced depth imaging (EDI) mode to assess the choroidal and retinal structures. Regarding the measurement of CBF using LSFG, the placement of the measurement circle at the macula in the affected eye was consistently adjusted throughout the follow-up period by referencing fundus photographs, OCT infrared images, and LSFG color map images.

**Figure 2:**
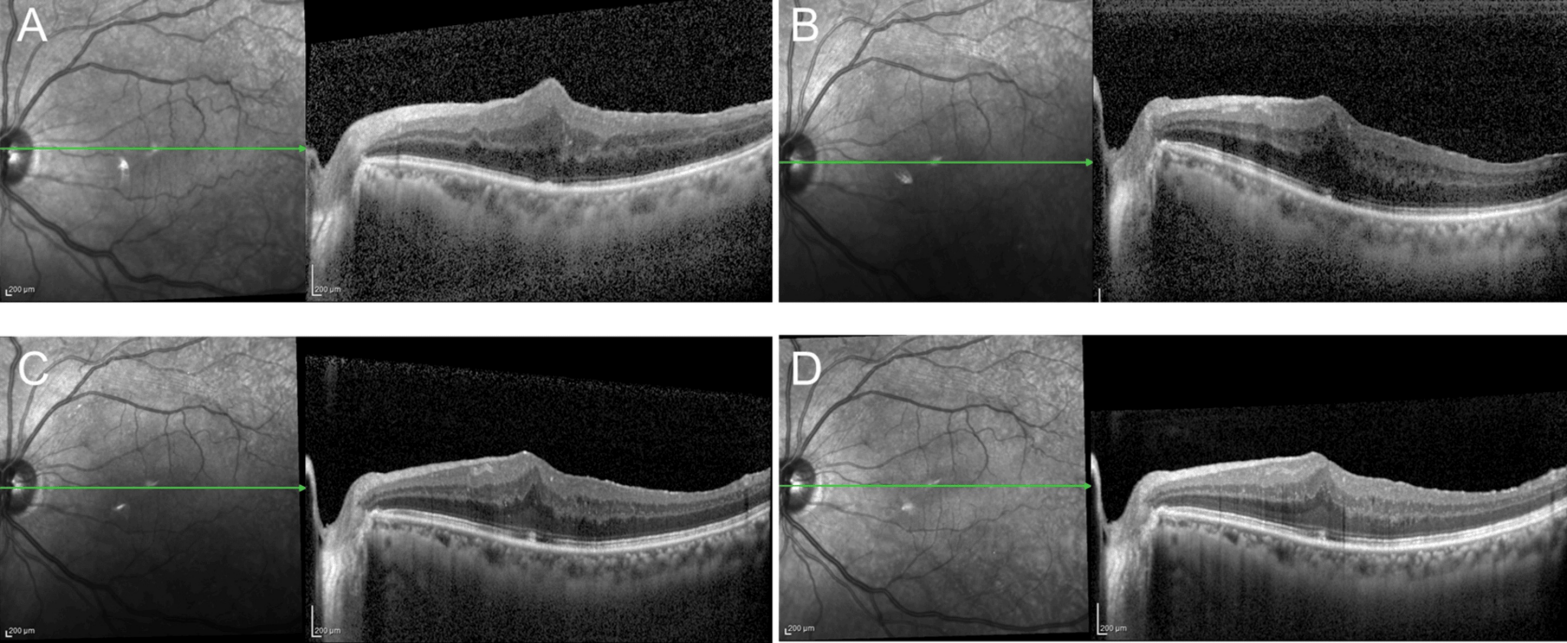
Optical coherence tomography findings after vitrectomy in Case 1 (A) One week: CCT of 396 μm and CFT of 614 μm. (B) One month: CCT of 190 μm and CFT of 561 μm. (C) Two months: CCT of 192 μm and CFT of 534 μm. (D) Three months: CCT of 187 μm and CFT of 514 μm. CCT: central choroidal thickness, CFT: central foveal thickness

**Figure 3:**
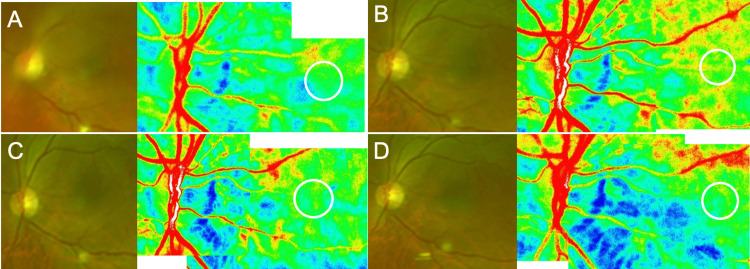
Laser speckle flowgraphy measurements of choroidal blood flow after vitrectomy in Case 1 (A) One week: 10.1 AU. (B) One month: 13.7 AU. (C) Two months: 11.3 AU. (D) Three months: 11.2 AU.

**Figure 4:**
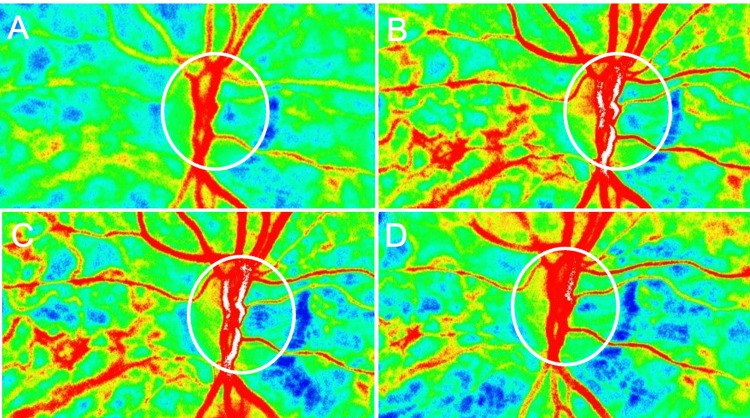
Laser speckle flowgraphy measurements of retinal blood flow (mean blur rate of vessel: MV) after vitrectomy in Case 1 (A) One week: 29.9 AU. (B) One month: 63.2 AU. (C) Two months: 60.3 AU. (D) Three months: 47.3 AU.

OCT showed that central choroidal thickness (CCT) was 396 µm, central foveal thickness (CFT) was 614 µm (Figure 2A), and LSFG findings revealed that the CBF was 10.1 arbitrary units (AU). The mean blur rate (MBR) in the vascular area (MV), representing retinal blood flow at the ONH, was 29.9 AU (Figure 3A, Figure 4A). One month after surgery, the BCVA improved to 20/29. Fundus examination revealed improvement in retinal hemorrhage, vitreous opacities, and fibrovascular membranes. OCT revealed a reduction in CCT to 190 µm and CFT to 561 µm (Figure 2B), while LSFG findings showed an increase in CBF to 13.7 AU and MV to 63.2 AU (Figure 3B, Figure 4B). Two months postoperatively, BCVA further improved to 20/22 with continued improvement in the fundus. OCT showed a CCT of 192 µm and a CFT of 534 µm (Figure 2C), while LSFG indicated a CBF of 11.3 AU and an MV of 60.3 AU (Figure 3C, Figure 4C). At three months, BCVA was 20/25, with no significant changes in the fundus examination. OCT showed a CCT of 187 µm and a CFT of 514 µm (Figure 2D), and LSFG findings indicated a CBF of 11.2 AU and an MV of 47.3 AU (Figure 3D, Figure 4D).

Ocular perfusion pressure (OPP) remained stable over time, with values of 54.2, 53.1, 53.6, and 55.3 mmHg at one week and one, two, and three months after vitrectomy, respectively. OPP was determined using the following formula: OPP = 2/3 (mean blood pressure) - IOP.

Case 2

A 70-year-old Japanese woman presented with worsening vision and ocular pain in her left eye that had persisted for one month. She had undergone suture-loaded intraocular lens implantation seven years earlier and was referred to our hospital with suspected chronic endophthalmitis.

**Figure 5:**
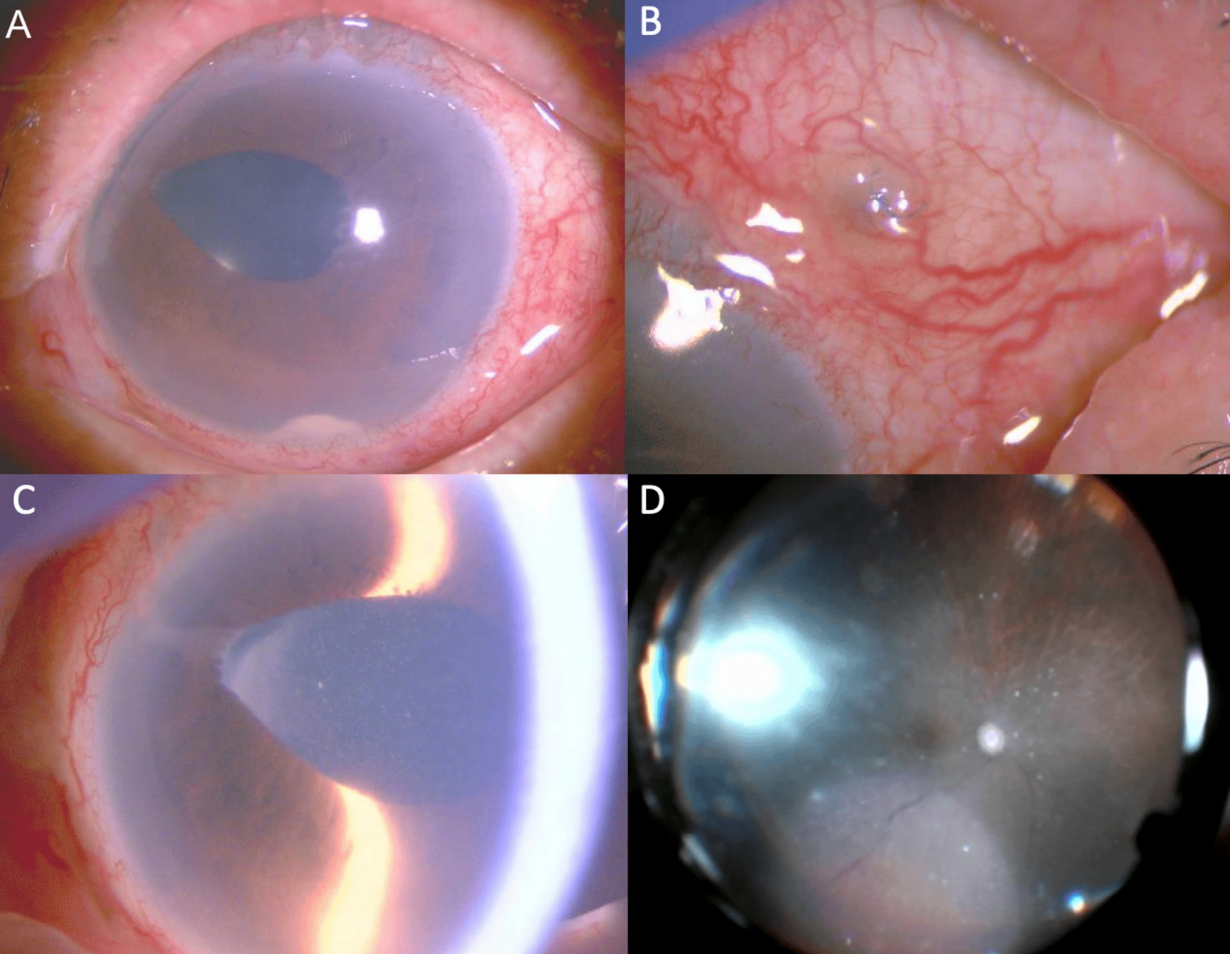
Clinical findings of postoperative endophthalmitis in Case 2 (A) Anterior segment photograph showing hypopyon and vitreous opacity. (B) Anterior segment photograph showing suture exposure from the previous intraocular lens fixation surgery, with mucus deposits observed around the exposed suture site. (C) Anterior segment photograph demonstrating significant anterior chamber inflammation, with residual vitreous prolapsed into the previous surgical wound at the side port. (D) Intraoperative fundus photograph during vitrectomy, showing retinal vascular leukocoria and retinal hemorrhage.

On initial examination, the BCVA was "hand motions" at 30 cm, and the IOP in the left eye was 13 mmHg. Slit-lamp examination revealed ciliary hyperemia, corneal edema, Descemet's membrane folds, suture exposure, anterior chamber opacities, and an intraocular lens. The fundus was opaque due to vitreous opacity (Figure 5A-5C).

The patient was diagnosed with chronic postoperative endophthalmitis. An emergency vitrectomy was performed, revealing vitreous opacity and retinal hemorrhage (Figure 5D). A culture of the vitreous fluid did not reveal the causative organism. Postoperatively, the patient received intravenous broad-spectrum antibiotics (imipenem/cilastatin 1 g twice daily) for one week. Additionally, antibiotic eye drops (moxifloxacin/cefepime) were administered for three months, and an antibiotic ointment (ofloxacin) was applied for one month. Unlike the severe postoperative inflammation in Case 1, which required systemic steroid administration, the milder inflammation in Case 2 was effectively managed with anti-inflammatory eye drops (betamethasone/bromfenac) for three months without needing oral corticosteroids.

**Figure 6:**
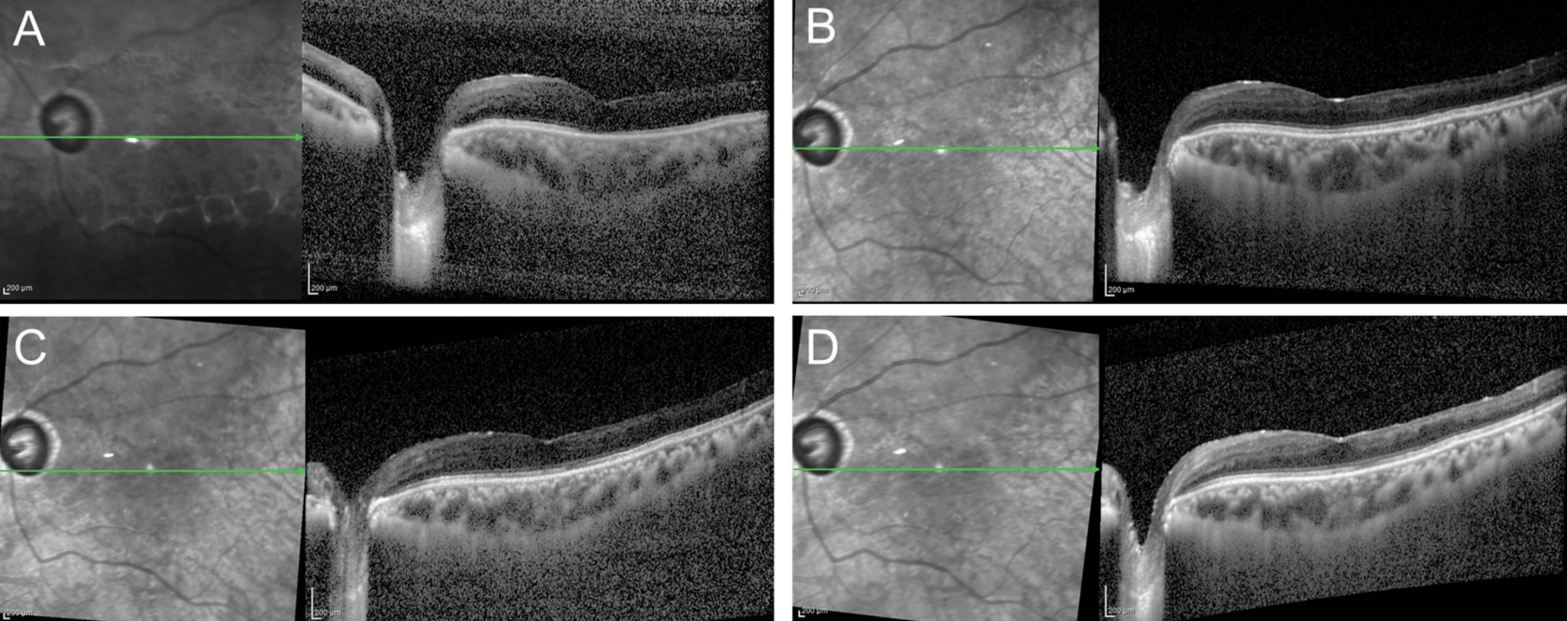
Optical coherence tomography findings after vitrectomy in Case 2 (A) One week: CCT of 396 μm and CFT of 223 μm. (B) One month: CCT of 380 μm and CFT of 240 μm. (C) Two months: CCT of 331 μm and CFT of 221 μm. (D) Three months: CCT of 333 μm and CFT of 234 μm. CCT: central choroidal thickness, CFT: central foveal thickness

**Figure 7:**
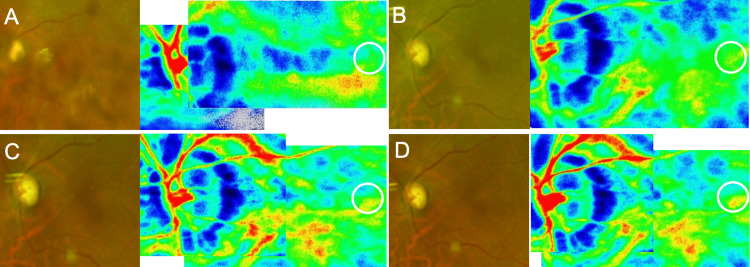
Laser speckle flowgraphy measurements of choroidal blood flow after vitrectomy in Case 2 (A) One week: 9.7 AU. (B) One month: 9.9 AU. (C) Two months: 11.2 AU. (D) Three months: 11.4 AU.

**Figure 8:**
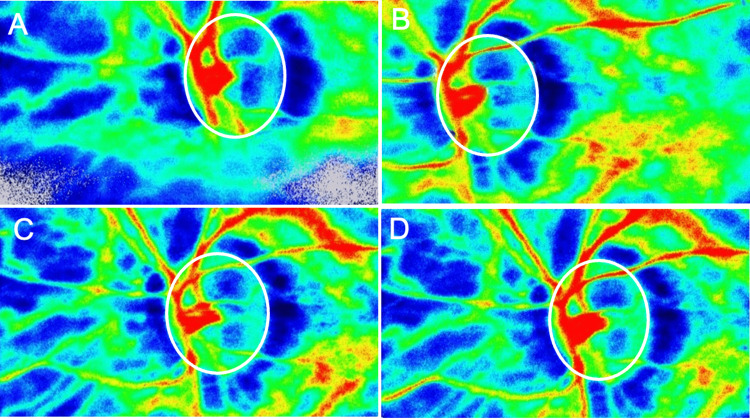
Laser speckle flowgraphy measurements of retinal blood flow (mean blur rate of vessel: MV) after vitrectomy in Case 2 (A) One week: 24.1 AU. (B) One month: 26.3 AU. (C) Two months: 24.9 AU. (D) Three months: 31.4 AU.

One week postoperatively, the BCVA was 20/67. Fundus examination revealed a retinal hemorrhage and vitreous opacity. Spectral-domain OCT showed a CCT of 396 µm and a CFT of 223 µm (Figure 6A), while LSFG findings indicated a CBF of 9.7 AU and an MV of 24.1 AU (Figure 7A, Figure 8A). One month postoperatively, BCVA improved to 20/40 with improved fundus findings. OCT showed a CCT of 380 µm and a CFT of 240 µm (Figure 6B), while LSFG indicated a CBF at 9.9 AU and an MV at 26.3 AU (Figure 7B, Figure 8B). Two months postoperatively, the BCVA remained at 20/40 with further fundus improvement. OCT showed a CCT of 331 µm and a CFT of 221 µm (Figure 6C), and LSFG indicated a CBF of 11.2 AU and an MV of 24.9 AU (Figure 7C, Figure 8C). Three months postoperatively, the BCVA was 20/33, with no significant changes in fundus examination. OCT showed a CCT of 333 µm and a CFT of 234 µm (Figure 6D), and LSFG indicated a CBF of 11.4 AU and an MV of 31.4 AU (Figure 7D, Figure 8D).

OPP remained stable over time, with values of 52.6, 50.0, 52.3, and 54.3 mmHg at one week and one, two, and three months post-vitrectomy, respectively.

## Discussion

These two cases demonstrate the recovery of ocular circulation following vitrectomy for postoperative endophthalmitis, which was quantitatively assessed using LSFG. This noninvasive technique provided real-time retinal and choroidal blood flow measurements, as reported in other ocular diseases [10-12]. Both cases showed progressive circulatory improvement correlated with reductions in CCT and improvements in visual acuity.

Bacteria causing endophthalmitis and their toxins may damage choroidal vessels by releasing cytolysins, hemolysins, and inflammatory cell wall components, disrupting the blood-ocular barrier and impairing perfusion [13]. Inflammatory cell infiltration into the choroidal tissue and blood vessels can damage vascular endothelial cells, leading to vascular occlusion and a subsequent reduction in CBF [1,14]. This reduction in CBF decreases macular perfusion and contributes to increased choroidal thickness due to inflammatory edema.

Vitrectomy plays a crucial role in managing these conditions by accelerating the intraocular transfer of antibiotics and facilitating the removal of inflammatory substances, thereby addressing the underlying infection [1,15]. Although a recent meta-analysis found no significant difference in visual outcomes between tap-and-inject (TAI) and pars plana vitrectomy (PPV) [15], it noted a potential selection bias, as more severe cases tended to undergo PPV. Therefore, we performed PPV due to the immediate availability of surgical resources, prioritizing rapid removal of infectious material to improve prognosis.

Furthermore, steroid treatment reduces choroidal inflammation, decreases choroidal thickness, and improves macular blood flow, ultimately enhancing overall choroidal circulation [16]. In the observed cases, postoperative LSFG measurements revealed a progressive decrease in CCT alongside an increase in CBF. This pattern reflects a reduced choroidal inflammation and improved choroidal circulation over time [17].

Additionally, the initial thickening of the choroid observed in these cases might be attributable to inflammatory cell infiltration and associated edema [13,18]. As inflammation subsides, the choroid becomes thinner and regains its functional capacity. The recovery of choroidal circulation enhances oxygen and nutrient supply to the outer retina, playing a critical role in supporting visual recovery.

Retinal blood flow, measured as MV by LSFG, is also affected by bacterial tissue invasion and toxins [19]. Inflammatory cell infiltration into retinal tissues and blood vessels can damage and occlude retinal vascular endothelial cells, leading to a reduction in retinal blood flow [1,14]. Similar to the choroidal changes, inflammatory edema initially increased retinal thickness, while MV was reduced. However, both vitrectomy and steroid treatments have been shown to improve retinal blood flow significantly. Vitrectomy facilitates the removal of inflammatory substances, while steroids provide anti-inflammatory effects and promote vasodilation, thereby enhancing retinal circulation [9,20]. As inflammation resolved following therapeutic intervention, LSFG measurements demonstrated progressive improvement in MV, which was accompanied by a gradual reduction in CFT. This relationship between blood flow recovery and decreased tissue edema suggests successful management of the inflammatory process in the retinal tissue.

This case has several limitations. First, baseline chorioretinal blood flow measurements before the onset of endophthalmitis were unavailable, making it unclear how much the blood flow was initially impaired and how much it improved postoperatively. Second, although most of the vitreous opacity was removed during vitrectomy, slight residual vitreous haze persisted at one week postoperatively, which may have influenced laser penetration during LSFG measurements. Third, as only one acute and one chronic case were analyzed, it remains uncertain how differences in blood flow recovery correlate with visual outcomes. Further studies with larger patient cohorts are necessary to validate these findings and establish the role of LSFG in managing postoperative endophthalmitis.

## Conclusions

In conclusion, these cases demonstrate the utility of LSFG in monitoring both retinal and choroidal blood flow during the treatment of postoperative endophthalmitis. The correlation between improved blood flow parameters and visual recovery emphasizes the importance of therapeutic interventions, including vitrectomy and steroid therapy, in restoring ocular circulation.
